# Cast in a racial and class mode of transport: exploring the “invisible” drivers of the minibus taxi industry in South Africa

**DOI:** 10.3389/fsoc.2026.1711861

**Published:** 2026-06-09

**Authors:** Siyabulela Christopher Fobosi

**Affiliations:** Department of Public and Constitutional Law, University of Fort Hare, Alice, South Africa

**Keywords:** employment precarity, informalization, labour relations, minibus taxi industry, South Africa

## Abstract

**Introduction:**

This paper analyses the precarious working lives of South African minibus taxi drivers and marshals/conductors, focusing on how their experiences intersect with broader debates on informalisation, precarity, and social justice. Drawing on Edward Webster's labour sociology and Karl Marx's analysis of veiled exploitation, the paper examines how labour within the taxi industry remains structurally invisible despite the sector's centrality to South Africa's public transport system.

**Methods:**

The study employed a qualitative research design grounded in a social constructivist epistemology. Data were collected through 102 semi-structured interviews and focus group discussions conducted in Johannesburg (2018-2020) and East London/Mdantsane (2012–2013). Additional data sources included participant observation and documentary analysis. Thematic analysis was used to identify recurring patterns related to labour precarity, institutional exclusion, and social reproduction.

**Results:**

The findings reveal deeply entrenched labour precarity characterised by the absence of formal employment contracts, commission-based remuneration through the “Target” system, excessive working hours, and exclusion from labour protections such as unemployment insurance and paid leave. Government interventions, particularly the Taxi Recapitalisation Programme (TRP) and Revised Taxi Recapitalisation Programme (RTRP), have prioritised vehicle modernisation while neglecting labour formalisation and worker protections. Workers also experienced weak representation, fragmented labour relations, and deteriorating conditions affecting household and social reproduction.

**Discussion:**

The paper argues that state interventions have produced redistribution without recognition, reinforcing existing inequalities rather than transforming labour conditions. Using Nancy Fraser's three-dimensional framework of social justice, the study demonstrates how the denial of recognition and representation perpetuates exploitation within the industry. The paper concludes that meaningful reform requires structural interventions aimed at formalising labour relations, enforcing employment protections, strengthening worker representation, and ensuring decent work within South Africa's minibus taxi industry.

## 1 Introduction

The minibus taxi industry is the lifeblood of South Africa's public transport system, carrying about 68% of daily commuters (Department of Transport, 2018). However, the sector is highly under-regulated and informalized with slave-like conditions of work. This paper aims to make the often “invisible” labor of the workforce, drivers and marshals taken into focus by investigating the highly precarious nature of their working existence (Burawoy, 2005). The sector's distinctive location of capital, race, and urban mobility—a “racial and class mode of transport”—has been a subject of scholarly study for some time (Clarke, 2006). Yet earlier work too readily spotlighted violence on-site, ownership relations, or regulatory anomalies, leaving the experience of workers in relative shade. To address this, my research offers a systematic sociological understanding of the working conditions at issue, grounded in a comprehensive body of empirical evidence gathered through qualitative interviews (Burawoy, 2008). Building on Webster's (2013) analysis of labor restructuring and the transformation of worker power in post-apartheid South Africa, the paper argues that these exploitative practices in the sector reflect a historical apartheid-based labor market combined with a contemporary regulatory regime that prioritizes the valorisation of capital for owners at the expense of workers' fundamental rights (Gorz, 1980) to support the idea of farewell to stable working-class employment.

The primary argument is that even though the government has made enormous efforts to formalize, through, for example, the Taxi Recapitalisation Programme (TRP) and its offspring, work conditions have not changed, as these projects misdiagnosed the problem: focusing on modernizing vehicles (redistributing of capital) and ignoring workers being formally recognized. This insight is captured by Fraser (2009): redistribution without recognition generates new injustices. The paper is organized as follows: Section 2 develops the theoretical framework, elaborating on precarious work, the precariat, and Nancy Fraser's three-dimensional conception of social justice (redistribution, recognition, representation) alongside Edward Webster's insights on labor dynamics under apartheid. Section 3 provides a revised historical and policy context, tracing the industry's evolution and presenting a timeline of key regulatory developments, including the Taxi Recapitalisation Programme (Department of Transport, 1999) and its successor, the Revised Taxi Recapitalisation Programme (Department of Transport, 2019). Section 4 details the qualitative research design, methodology, and analytical approach. Section 5 presents the empirical findings organized thematically, with analysis linking to the conceptual framework. The final section concludes with implications for policy and future research.'

## 2 Theoretical framework

Labor relations in South Africa's minibus taxi industry are notoriously opaque and ungoverned (Webster, 1990). The vast majority of drivers and marshals are thought to be classed as “casual workers.” Rather, they work on casual, ad-hoc relations with owners- the so-called “commission system” (Fobosi, 2021). Drivers must hand over a fixed daily payment, known as “Target”, to the owner and retain whatever is left over as their commission. This system neatly shifts all risk of operation from the owner (capital) onto the driver (labor) (Webster and Macun, 1998). Such extreme exploitation is the basis of the chronic precarity that workers face.

The conceptual anchoring of this study rests on three interrelated theoretical pillars: Karl Marx's analysis of veiled exploitation in the labor process, Guy Standing's conceptualization of the precariat, and Nancy Fraser's multi-dimensional framework of social justice. Each offers distinct yet complementary analytical tools for understanding the condition of taxi workers.

Marx's (1890) analysis of the capitalist labor process reveals how the appearance of a free contractual relationship between employer and employee obscures the extraction of surplus value. In the minibus taxi industry, this veiled process is starkly evident: the commission system, wherein drivers surrender a fixed daily “Target” to owners, formally appears as a partnership arrangement. Substantively, however, it transfers all operational risk onto drivers while owners retain secure asset ownership. This represents what Marx termed the “hidden abode of production”, where the apparent equality of exchange masks fundamental exploitation. The labor power purchased by owners is transformed into value only through drivers' extended working days, which regularly exceed 15 h, yet this value is disproportionately appropriated as owner profit rather than driver remuneration (O'Doherty and Willmott, 2009; Edwards, 2010; Spencer, 2000).

Standing's (2011) concept of the precariat advances this analysis by specifying the multidimensional insecurities characterizing contemporary labor. The precariat is distinguished by the absence of seven forms of labor security: labor market security (adequate income-earning opportunities); employment security (protection against arbitrary dismissal); job security (ability to retain specified niches in employment); work security (protection against accidents and illness); skill reproduction security (opportunities to gain and retain skills); income security (assurance of adequate stable income); and representation security (collective voice in labor markets) (see also Weiss, 2005). Taxi workers in this study exemplify this condition: they lack employment contracts (thus no employment security), face dismissal without recourse (no job security), endure extended hours without overtime (no work security), receive no training (no skill reproduction security), experience fluctuating earnings dependent on daily passengers (no income security), and are systematically excluded from union representation (no representation security). Recent developments, however, suggest that even within highly precarious sectors, forms of associational power may begin to emerge. (Webster and Dor 2023) argue that new worker organizations are increasingly attempting to organize fragmented and informalized labor, although their effectiveness remains contingent on supportive institutional and regulatory frameworks. Unlike the salariat, who retain employment-based entitlements, taxi workers are, in Standing's terms, “denizens” rather than full citizens of the labor market (Warhurst, 1998).

Fraser's (2009) framework of social justice provides the analytical architecture for evaluating state interventions in the industry. Fraser argues that justice requires three irreducible dimensions: redistribution (economic arrangements ensuring equitable resource allocation), recognition (cultural-institutional arrangements respecting parity of participation), and representation (political arrangements ensuring voice in decision-making). These dimensions are mutually constitutive: misrecognition—the denial of workers' status as rights-bearing subjects—reinforces maldistribution—the channeling of resources to owners rather than workers. Critically, Fraser insists that justice claims cannot be reduced to any single dimension. The TRP's exclusive focus on vehicle modernization represents what Fraser terms “affirmative” redistribution—correcting inequitable outcomes without disturbing the underlying social structures generating them. What remains absent is “transformative” recognition: acknowledging taxi workers as legitimate labor subjects entitled to the full protections of employment law. This theoretical synthesis—Marx's exposure of veiled exploitation, Standing's specification of precarity's dimensions, and Fraser's three-dimensional justice framework—guides the analysis that follows.

## 3 Historical context and policy landscape

This section traces the industry's evolution across four significant periods: 1977–1987, 1987–1994, 1994–1999, and 1999–2018. Drawing on Webster's (1985) analysis of labor dynamics under apartheid, we see how the taxi industry emerged as a site of black economic resistance and entrepreneurship. However, the industry's informal nature and lack of regulation have perpetuated precarious working conditions and labor exploitation. The post-apartheid period saw efforts to formalize the industry through initiatives like the Taxi Recapitalisation Programme (TRP), but these have had mixed results (South African Transport and Allied Workers Union, 2012). By contextualizing the industry's history, we can better understand the ongoing challenges and the need for inclusive policy interventions. The minibus taxi industry, predominantly founded by the black community, emerged as a crucial transport system primarily serving this demographic (Baloyi, 2012). Its' roots trace back to the 1930s in areas like Natalspruit (now Katlehong), where taxis, mainly sedans, were permitted to carry up to four passengers (Godsell, 2016). During apartheid, black people faced severe restrictions on entering the industry due to segregation laws and financial constraints. These laws and the “One-Bantu-One-Business” policy limited black individuals to owning a single business, making it nearly impossible for them to operate taxis (Fourie, 2003).

Despite these challenges, the period from 1977 to 1987 saw the minibus taxi sector striving for recognition as a legitimate public transport operator. Initially, sedans like Valiants and Chevrolets were used, but by 1977, operators began using minibuses with ten seats, known as kombi taxis (Venter, 2013). The 1977 Road Transportation Act, influenced by the Breda Commission's recommendations, proposed deregulation, allowing for freer trade and less regulation in the minibus taxi industry (Nkambule and Govender, 2014; Gibbs, 2014).

The period from 1987 to 1994 marked significant changes due to the deregulation process initiated in 1987. This deregulation allowed more black operators to enter the industry, transforming it into a vehicle for black capital accumulation. During this time, taxi violence and illegal operations without licenses became rampant. Despite these issues, the industry's demand grew, and the government's reluctance to grant licenses led to significant tension (Barrett, 2003). The Welgemoed Commission of 1983 had suggested making minibus taxis illegal by limiting licenses, but by 1989, a limited number of permits were issued, allowing the industry to expand rapidly (Ingle, 2009). By 1986, 16-seaters were legalized for taxi use, further propelling growth. This era saw the minibus taxi industry becoming a dominant mode of transport, viewed by many as a community-based sector that resisted apartheid without government subsidies (Sekhonyane and Dugard, 2004). The historical development of the minibus taxi industry reflects shifting regulatory regimes and political-economic conditions that shaped both ownership structures and labor relations. Table 1 summarizes the key historical phases and regulatory developments that structured the evolution of the sector, highlighting how deregulation, post-apartheid policy reforms, and more recent recapitalisation initiatives created the institutional context within which contemporary labor precarity emerged.

**Table 1:** Historical development of South Africa's minibus taxi industry.

Period	Key developments	Regulatory context
1930–1976	Sedan taxis operate in townships; black operators excluded by ‘One-Bantu-One-Business' policy	Apartheid segregation; Urban Areas Act (1945) restricts black business
1977–1986	Ten-seater minibuses introduced; Road Transportation Act (1977) enables deregulation	Breda Commission recommends freer competition
1987–1994	Full deregulation; taxi violence escalates; 16-seaters legalized (1986)	Transport Deregulation Act (1988)
1995–1999	National Taxi Task Team (NTTT) recommends formalization	Transition to democracy; new constitutional framework
1999–2018	Taxi Recapitalisation Programme (TRP) introduced; scrapping of old vehicles	National Land Transport Transition Act (2000)
2019–2025	Revised Taxi Recapitalisation Programme (RTRP); Competition Commission Inquiry (2020); COVID-19 impacts	Sectoral Determination 11; National Land Transport Act (2009)

Source: Author's design.

As Table 1 illustrates, policy interventions have historically focused primarily on industry regulation and vehicle modernization, with comparatively little attention paid to labor relations and workers' protection. The post-apartheid period brought new challenges and opportunities. The 1994 general election led to an increase in taxi-related violence, as the industry, previously heavily regulated, experienced a shift toward deregulation, resulting in more decentralized and criminal activities (Fourie, 2003). Efforts to formalize the industry began with the establishment of the Taxi Recapitalisation Programme (TRP) in 1999, aimed at stabilizing and modernizing the sector (Mahlangu, 2002). Since 1999, the government has focused on reforming the minibus taxi industry through initiatives like the TRP and the Revised TRP. The National Land Transportation Transition Act (NLTTA) of 2000 aimed to restructure land transport, enhancing the efficiency and safety of public transport (Competition Commission, 2020). The minibus taxi industry, with a 65% market share in public transport, continues to be a vital link between the formal and informal economies.

The introduction of the Bus Rapid Transit (BRT) system, such as Rea Vaya in Johannesburg, marked a significant development. Negotiations starting in 2009 led to the inclusion of minibus taxi operators in the BRT scheme, ensuring their participation by removing their vehicles from competing routes (McCaul and Ntuli, 2011). This integration aimed to formalize and modernize the industry while addressing long-standing issues like road safety and operator disputes.

Research on South Africa's minibus taxi industry has extensively documented the regulatory frameworks and policies shaping its development (Baloyi, 2012; Fourie, 2008; Imaniranzi, 2015; Gibbs, 2014; Neumann et al., 2015). However, there remains a critical gap in empirical evidence concerning the precariousness of work within the industry, particularly in relation to the Taxi Recapitalisation Programme (TRP) and its broader impact on working conditions, such as the quality of taxis used for transport. Eddie Webster's theoretical insights on labor dynamics and social reproduction provide a valuable framework for understanding the impact of policies like the TRP on workers within the minibus taxi industry. Webster's work emphasizes the relationship between wage labor and capital, examining how economic and social structures influence working conditions and labor power reproduction (Von Holdt and Webster, 2005; Jonna and Foster, 2016). In the context of the minibus taxi industry, the poor quality of many taxis directly impacts drivers' ability to sustain their labor power. Driving older and poorly maintained vehicles not only affects their safety but also compromises their earning potential and overall job satisfaction (Von Holdt and Webster, 2005).

The period 2018–2025 has witnessed intensified contestation over the industry's future, alongside significant policy developments. In 2019, the Department of Transport formally launched the Revised Taxi Recapitalisation Programme (RTRP), increasing the scrapping allowance from R91,100 ($5,650) to R124,000 ($7,690) per vehicle and introducing provisions for collaborative ownership models, including cooperatives and corporatised entities. The RTRP explicitly acknowledged, for the first time, the need to address working conditions, with stated objectives including “the provision of decent and secure employment to employees of the taxi industry” (Department of Transport, 2019). However, implementation has been uneven. By March 2023, approximately 89,000 old taxi vehicles had been scrapped nationally, yet the Department of Employment and Labour's inspections revealed persistent non-compliance with Sectoral Determination 11, with fewer than 15% of taxi operators maintaining formal employment contracts for drivers (Department of Employment and Labour, 2023).

The Competition Commission's Market Inquiry into Competition Commission South Africa (2020) provided the most comprehensive official examination of the industry to date. The Inquiry found that the minibus taxi sector employs approximately 600,000 people directly, including 300,000 drivers, 100,000 rank marshals, and 100,000 car washers, with an additional 150,000 informal traders dependent on taxi rank economies. Crucially, the Inquiry confirmed that “the commission system, whereby drivers pay owners a fixed daily amount and retain the surplus, remains the dominant remuneration model, despite being incompatible with the Basic Conditions of Employment Act” (Competition Commission, 2020, p. 245). The Inquiry recommended the phased introduction of minimum wages linked to formal employment contracts, yet progress has been limited.

The COVID-19 pandemic (2020–2022) exposed and amplified the precarity of taxi workers. During national lockdowns, minibus taxis were classified as essential services, yet drivers received no government income support, as the Unemployment Insurance Fund (UIF) excluded those without formal contracts. Research conducted during this period found that 78% of drivers experienced food insecurity, and 62% accrued debt to meet daily “Target” payments to owners during periods of reduced passenger demand (Fobosi, 2023). The pandemic thus crystallized the structural injustice Fraser's framework illuminates: workers essential to urban mobility were rendered invisible to social protection systems precisely because they lacked recognition as formal employees.

## 4 Methodology

### 4.1 Research design and questions

This study employs a qualitative research design grounded in a social constructivist epistemology, seeking to understand how taxi workers themselves construct meaning around their precarious working conditions (Blanche et al., 2006). The research was guided by the primary question: *To what extent has the Taxi Recapitalisation Programme (TRP) affected precarious working conditions within South Africa's minibus taxi industry?* Subsidiary questions explored: (i) How do workers experience the commission-based remuneration system? (ii) What forms of labor (in)security characterize employment in the industry? (iii) How do state interventions address (or fail to address) workers' recognition and representation claims? (Neuman, 2003).

### 4.2 Research sites and sampling

Data collection occurred across two phases and two sites. Phase one (2012–2013) was conducted in East London and Mdantsane, Eastern Cape, and involved 44 participants, and was part of the Master of Arts in Industrial Sociology Degree at Rhodes University. Phase two (2018–2020) was conducted in Johannesburg, Gauteng, and comprised 58 participants, and was for the Doctor of Philosophy in Industrial Sociology Degree at the University of Johannesburg. The two-site design enables comparison between a secondary city with deep historical ties to homeland labor migration (East London/Mdantsane) and the primary economic hub where formalization policies have been most intensively implemented (Johannesburg). Sampling was purposive: participants were recruited through taxi associations, rank-based snowballing, and direct approach at ranks during off-peak hours. Inclusion criteria required minimum 6 months' experience in the industry. The final sample of 102 participants comprised 41 taxi drivers, 18 taxi marshals, 22 taxi owners, and 21 key informants (government officials, trade union representatives, industry body officials) (Buffalo City Metropolitan Municipality, 2011–2016; Buffalo City Municipality, 2005).

### 4.3 Data collection methods

Four methods were employed. First, semi-structured interviews explored employment relationships, remuneration systems, working hours, access to social protections, and experiences of dismissal. Interviews averaged 45–75 min and were conducted in isiZulu, isiXhosa, Sesotho, or English according to participant preference. Second, three focus group discussions (one in Mdantsane, two in Johannesburg) with 6–8 drivers each explored collective experiences and enabled participants to challenge and refine emerging interpretations. Third, participant observation was conducted at four Johannesburg taxi ranks (Wanderers, Noord, Faraday, Bree) and three East London ranks, involving 45 h of observation documenting daily routines, interactions between marshals and drivers, and negotiation of rank access. Fourth, documentary analysis examined TRP policy documents (2007), RTRP frameworks (2019), Department of Transport annual reports, and Competition Commission inquiry transcripts (Janesick, 1998).

### 4.4 Thematic analysis process

Analysis followed Braun and Clarke's (2006) six-phase approach. Phase 1: familiarization through repeated transcript reading and observational note review. Phase 2: systematic open coding using ATLAS.ti software (version 8), generating 147 initial codes. Phase 3: code clustering into potential themes, iteratively refined through comparison with theoretical concepts from Standing and Fraser. Phase 4: theme review against coded extracts and full dataset. Phase 5: theme definition and naming, resulting in four analytical themes: (i) entrenched labor precarity and informalization impasse; (ii) weak institutional protection; (iii) ambivalent internal labor relations; (iv) social reproduction implications. Phase 6: report writing, with themes mapped to Fraser's justice dimensions: redistribution (Theme 1), recognition (Themes 2 and 3), representation (Theme 2). Analysis was both inductive (allowing themes to emerge from data) and deductive (interrogating data through theoretical lenses).

### 4.5 Ethics and positionality

Ethical approval was obtained from the University of Johannesburg (REC-02-156-2017) and Rhodes University Faculty of Humanities Higher Degrees Committee. Due to participants' constant mobility and documented industry intimidation, verbal informed consent was obtained after full explanation of research purposes, risks, and confidentiality protections. A secure consent log was maintained separately from data. All names are pseudonyms; locations are identified only by city and role. As a black South African researcher conversant in participants' home languages, I occupied an “insider-outsider” position: insider through shared linguistic and racialised experience of South African spatial inequality; outsider through academic location and non-participation in daily rank labor. Reflexive field notes documented how this positionality shaped data collection, including moments when participants tested my trustworthiness through questions about research benefits and potential repercussions (Patnaik, 2013; Bourdieu et al., 1963).

## 5 Findings and discussion

Thematic analysis identified four interconnected dimensions of precarious working life, each linked to Fraser's justice framework: entrenched labor precarity (redistribution failure), weak institutional protection (recognition failure), ambivalent internal labor relations (recognition and representation failures), and implications for social reproduction (the embodied consequences of all three failures). Across the 102 participants, systematic patterns emerged.

### 5.1 Labor precarity and informalization impasse

The absence of formal employment contracts constituted the defining feature of work in the industry. Of 59 workers interviewed, 57 (96.6%) reported having no written contract with the taxi owner. This absence rendered all aspects of employment relationships discretionary and revocable. A driver at Faraday Taxi Rank explained:

“*We have no job security. If the owner wants to fire us, there's no recourse for us. We don't even have contracts. You can work for five years, then one morning the owner says ‘bring the keys, find another taxi,' and that's it. No hearing, no notice, nothing.”* (Taxi Driver, Johannesburg, Faraday Rank)

This absence of contractual protection was compounded by the commission-based “Target” system, which transferred all operational risk onto drivers. Drivers reported daily payments to owners ranging from R400–R800 ($25–$50) depending on route profitability. Analysis of income data from 41 drivers revealed average monthly earnings of R4,200–R5,800 ($260–$360), placing most below the R3,413 ($211), when calculated on a 48-h week basis—yet drivers worked an average of 72 h weekly, meaning effective hourly rates fell substantially below the statutory minimum. A driver in East London quantified the arrangement:

“*The owner wants R600 ($37) every day. If I make R1,000 ($62) I keep R400 ($25). If I make R700 ($43) I keep R100 ($6). So you keep driving, hoping for better days, but the owner never loses. He gets his money whether I eat or not.”* (Taxi Driver, East London)

This system exemplifies Standing's precariat condition: income insecurity (fluctuating daily earnings), employment insecurity (dismissal without cause), and work insecurity (extended hours without overtime). Critically, the TRP and RTRP left this structure untouched. Despite government expenditure of over R6 billion ($372 million) on vehicle scrapping between 2006 and 2023, no policy document required operators to convert drivers to formal employment as a condition of participation. As Fraser's framework predicts, redistribution without recognition—channeling capital to owners while denying workers' status as rights-bearing subjects—perpetuated rather than remedied injustice (Paret, 2013).

### 5.2 Weak institutional protection: the recognition failure

Workers' exclusion from labor law protections was systematic. Of 59 workers, none were registered for Unemployment Insurance Fund (UIF) contributions; none received paid annual leave or sick leave; none had pension or provident fund arrangements (Employment Conditions Commission, 2012). This exclusion was not merely de facto but actively maintained through institutional practices. A Department of Employment and Labor inspector acknowledged the enforcement gap:

“*We have 25 offices in Gauteng, but the taxi industry... we can't just walk into a taxi rank like we walk into a factory. There's no register, no gate, no manager's office. The drivers are moving constantly. The owners are often not there. And if we try to inspect, we face hostility – drivers afraid to talk, marshals who see us as trouble. The sectoral determination exists on paper, but making it real in this industry...”* (Department of Employment and Labour official, Johannesburg)

This institutional invisibility constitutes what Fraser terms misrecognition: workers are not recognized as legitimate labor subjects entitled to protection. The consequences were starkly illustrated during COVID-19, when drivers—classified as essential workers—were excluded from UIF relief because they had not paid contributions. A marshal in Soweto described the experience:

“*When lockdown came, we kept driving. Essential service, they said. But when we needed help – when passengers disappeared and we couldn't make the daily Target – the government said we don't exist. No UIF, no relief. The owner still wanted his money. We drove empty taxis just to show him we were trying.”* (Taxi Marshal, Johannesburg)

Quantitative analysis of institutional protection revealed: 0% of workers had formal employment contracts; 0% were registered for UIF; 0% received paid leave; 92% reported experiencing arbitrary dismissal or threat of dismissal in the preceding 3 years; 78% reported working 70+ h weekly.

### 5.3 Ambivalent internal labor relations

Relations between owners, drivers, and marshals were characterized by what Edwards (2018) terms “structured antagonism”—conflict embedded in the organization of work itself. The commission system positioned marshals as intermediaries between owners' revenue demands and drivers' exhaustion. A marshal in Johannesburg described this dilemma:

“*The marshal is damned if he does, and damned if he doesn't. The owner wants their money by 6 PM, no excuses. The driver is exhausted, wants a rest, maybe hasn't eaten all day. The association demands order in the rank. If I push the driver too hard, he hates me. If I don't get the money, the owner curses me. There's no right way to do this job.”* (Taxi Marshal, Johannesburg)

This structure fragmented worker solidarity. Drivers competed for passengers within ranks, undermining collective action. Union organizers from SATAWU reported extreme difficulty recruiting members:

“*Intimidation is massive. We recruit a driver, he agrees, then next week his owner says ‘I heard you met with the union.' Suddenly he's working night shift only, or his taxi is sent to a dangerous route. We lose him. The owners know that if drivers organise, the whole Target system collapses. So they prevent it – sometimes with threats, sometimes with violence.”* (SATAWU organiser, Johannesburg)

Representation security—Standing's seventh form—was thus systematically denied. None of the 41 drivers interviewed were union members; 85% expressed fear that joining a union would result in dismissal. However, this finding must be interpreted within a shifting institutional context. Recent scholarship by Webster and Dor (2023) highlights the emergence of new forms of associational power among precarious workers, even within highly fragmented and informalized sectors. In the South African minibus taxi industry, this is reflected in the formation of the South African Taxi Drivers and Allied Workers Union (SATDWU), which is affiliated with the Congress of South African Trade Unions (COSATU).

The existence of SATDWU signals an important, albeit nascent, development toward formalized worker representation in a sector historically characterized by its absence. Nevertheless, the findings of this study indicate that such associational power remains limited in practice. The structural features of the industry—including the spatial mobility of workers, the absence of fixed workplaces, and the dominance of the commission-based “Target” system—continue to undermine sustained union organization. As Webster and Dor (2023) argue, associational power in precarious sectors is often emergent but fragile, requiring enabling institutional conditions to become effective.

In this respect, the absence of union membership among participants should not be read as a complete absence of worker organization, but rather as evidence of the uneven and contested consolidation of associational power within the taxi industry. Without stronger legal protections and enforcement mechanisms to secure freedom of association, these emerging forms of worker organization are unlikely to significantly alter the balance of power between labor and capital.

### 5.4 Implications for social reproduction

Precarity extended beyond the workplace into workers' lives and households. The concept of social reproduction—the daily and generational processes sustaining labor power—illuminates how exploitation in production undermines life outside production. Drivers' extended hours (average 15 h daily, 6–7 days weekly) precluded participation in domestic life, childcare, and community. A driver in East London described the impossibility of maintaining family relationships:

“*I leave home at 4 AM. My children are sleeping. I return at 9 PM. They are sleeping again. Weekends I work because that's when people travel most. My wife says I'm a stranger in my own home. She's right. But if I stop one day, the Target isn't met, and the owner finds another driver – there are hundreds waiting. So I keep driving.”* (Taxi Driver, East London)

This testimonial illuminates the embodied consequences of Fraser's three-dimensional injustice. Redistribution failure (inadequate, insecure income) prevents saving or household investment. Recognition failure (denial of worker status) excludes drivers from social protections that might buffer against illness or injury. Representation failure (absence of collective voice) leaves structural exploitation unchallenged. The result is what one driver termed “living death”—labor that sustains owners' accumulation while exhausting workers' capacity to sustain themselves or their families.

Analysis of health impacts among 41 drivers revealed: 73% reported chronic back or joint pain; 68% reported untreated hypertension; 62% reported urinary tract infections from inadequate toilet access; 59% reported symptoms of depression or anxiety. These figures underscore that precarity is not merely an employment category but a condition that actively destroys workers' physical and social reproduction capacity.

### 5.5 Redistribution without recognition: the racialised contradiction of state intervention

The state's interventions exemplify Fraser's central insight: redistribution unaccompanied by recognition generates new injustices. The TRP and RTRP directed substantial public resources—over R6 billion in scrapping allowances alone—to taxi owners, facilitating vehicle modernization. Yet these programmes systematically excluded workers from their scope. No TRP document mentions drivers or marshals as beneficiaries; no condition required improved wages or contracts; no monitoring assessed working conditions. A Department of Transport official acknowledged this selectivity:

“*The programme's target was the vehicle and the operator. If the vehicle is safe, if the operator is compliant with licensing*,

*we've achieved our objective. Labour conditions... that falls to the Department of Employment and Labour. Different mandate.”* (Department of Transport official, Pretoria)

This bureaucratic compartmentalisation masks a substantive choice: to treat capital (vehicles) as the object of intervention while rendering labor invisible. The consequence is that new, publicly subsidized, safer vehicles become sites for the continuation of old exploitation. Drivers now perform grueling 15-h shifts in R400,000 vehicles under identical verbal, commission-based arrangements to those prevailing two decades ago. A driver in Johannesburg articulated this contradiction:

“*The owner got R124,000 ($7,690) from government to scrap his old taxi. He bought a new Quantum with a loan. Now I drive a beautiful taxi – gair conditioning, comfortable seats. But I still pay R650 daily Target. I still have no contract. I still get no UIF. The taxi is new; my life is the same.”* (Taxi Driver, Johannesburg)

This pattern exemplifies what Webster (2005) identified as South Africa's capacity to absorb modernity—new technology, new vehicles—without transforming underlying social relations. The racialised dimension remains central: the industry was built by black entrepreneurs excluded from formal economy; state intervention now reinforces class differentiation within the black community, with owner accumulation predicated on worker exploitation. Fraser's framework reveals that recognition—acknowledging drivers as legitimate labor subjects entitled to protection—is the precondition for just redistribution. Until policy sees workers, not only vehicles, capital subsidies will continue to entrench rather than remedy precarity (Webster, 1973, 2022; Webster and Pampallis, 2017; Bezuidenhout et al., 2022).

## 6 Conclusion

This paper has analyzed the precarious working lives of minibus taxi drivers and marshals in South Africa through the integrated theoretical lens of Marx's veiled exploitation, Standing's precariat, and Fraser's three-dimensional justice framework. The evidence demonstrates that entrenched precarity—characterized by contract absence, commission-based risk transfer, extended hours, and institutional invisibility—constitutes the industry's foundational labor regime. State formalization interventions, notably the TRP and RTRP, have not merely failed to address this regime but have actively reinforced it by channeling public resources to owners (redistribution) while denying workers' status as rights-bearing subjects (recognition) and excluding them from policy processes (representation).

Four key findings advance scholarly understanding. First, the commission ‘Target” system exemplifies Standing's precariat condition: workers bear all operational risk while owners retain asset security, producing income insecurity, employment insecurity, and work insecurity simultaneously. Second, institutional protection is systematically absent: 96.6% of workers lack contracts, 0% access UIF or paid leave, and 92% experience arbitrary dismissal threats—a pattern constituting Fraser's misrecognition, where workers are rendered invisible to labor law. Third, internal labor relations are structured by antagonism that fragments worker solidarity and precludes collective voice, denying representation security. Fourth, precarity's consequences extend beyond employment to destroy workers' capacity for social reproduction—sustaining families, maintaining health, participating in community—thereby perpetuating intergenerational disadvantage.

Fraser's framework proves essential for diagnosing state failure. The TRP/RTRP's exclusive focus on vehicle modernization represents redistribution without recognition: capital subsidies to owners accompanied by systematic denial of workers' personhood as labor subjects. This selectivity is not inadvertent but structural, reflecting what Webster identifies as apartheid's labor market legacy: a racialised distinction between those entitled to protection and those consigned to informalized exploitation. The result is that new, publicly subsidized vehicles become sites for old exploitation—an instance of South African modernity absorbing technology while preserving social relations.

The paper makes three contributions. Empirically, it provides the most detailed worker-centered analysis of taxi industry precarity to date, drawing on 102 interviews across two cities. Theoretically, it synthesizes Marx, Standing, and Fraser to specify precarity's multiple dimensions and demonstrate their interconnection. Methodologically, it demonstrates how qualitative research can illuminate the “hidden abode” of informalized labor, rendering visible processes usually obscured.

Recent developments, such as the emergence of the South African Taxi Drivers and Allied Workers Union (SATDWU), point to the possibility of building associational power within the sector. However, in the absence of structural reforms that protect workers' rights to organize and shield them from employer retaliation, such initiatives remain limited in their capacity to transform labor relations in the industry. Policy implications are urgent. Effective intervention requires the state to assume its constitutional responsibility to enforce standard employment contracts, establish a minimum wage that prohibits Target-based exploitation, and ensure comprehensive social security access for all taxi workers. This demands inter-departmental collaboration: the Department of Transport must condition future recapitalisation funding on operators' compliance with labor law; the Department of Employment and Labor must develop rank-based enforcement strategies; the Department of Home Affairs must address the vulnerabilities of migrant drivers. Without such measures, the “racial and class mode of transport” will continue reproducing the injustice Fraser identifies: redistribution that entrenches rather than remedies inequality because it denies those it purports to help recognition as equal participants in social life.

## Data Availability

The datasets generated and analyzed during the current study are not publicly available due to confidentiality and ethical restrictions protecting participant anonymity but may be made available by the author on reasonable request.
